# How do I recognise and manage visual snow syndrome?

**DOI:** 10.1038/s41433-024-03059-4

**Published:** 2024-04-16

**Authors:** Clare L. Fraser

**Affiliations:** 1 Save Sight Institute, Faculty of Health and Medicine, The University of Sydney, 8 Macquarie Street, Sydney, NSW 2000 Australia; 2 Macquarie Ophthalmology, School of Clinical Medicine, Macquarie University, Sydney, NSW Australia

**Keywords:** Eye manifestations, Diagnosis

## Abstract

Visual snow syndrome (VSS) is becoming increasingly recognised by clinicians and self-diagnosed by our patients thanks to online search tools. Previously this phenomenon was overlooked or dismissed leading to increased patient anxiety. Now, however, we need to be careful with that we are not making what would seem to be an easy diagnosis of VSS when actually we are missing any underlying or mimic conditions.

## Abstract (zh)

由于线上检索工具的发展, 视雪症 (VSS) 正日益被临床医生所认识, 并被患者自我诊断。以前, 这种现象经常被忽视或否认, 导致焦虑的患者增加。然而, 需要小心的是, 我们不能仅仅因为VSS看似简单就轻易进行诊断, 实际上可能忽视了其他潜在的或类似的疾病。

## Introduction

In visual snow syndrome (VSS) patients report seeing uncountable, dynamic, tiny dots flickering continuously across the entire visual field of both eyes. They describe this as being like the black-and-white static or the ‘snow’ on a poorly tuned analogue television. While VSS could be thought of as a hallucination because there is no real-world correlate of the perception, it may be more accurate to consider it to be an illusion created by disordered visual processing. This hypothesis is supported by the presence of other visual phenomena that these patients often describe; persistence of afterimages, trail phenomena, sensitivity to bright light or night blindness and an increased awareness of normal entoptic phenomena (visual perceptions whose source is within the eye itself, for e.g., floaters) [1]. The pathophysiology of visual snow is still under investigation.

A diagnostic framework for VSS has been published which aims to capture the range of symptoms patients describe with this condition (Table 1) [2]. The diagnosis of VSS can be made when a patient presents with subjective black-and-white visual static with at least one associated symptom of palinopsia (abnormal persistence or recurrence of an image in time), photopsia (flashes of light), nyctalopia (difficulty seeing in dim light or night-time) and entoptic phenomena. The diagnosis excludes those that have a history more consistent with migraine aura or the symptoms have occurred secondary to illicit drug use.

**Table 1:** Proposed criteria for visual snow syndrome [2].

1	Visual snow: dynamic, continuous tiny dots in the entire visual field lasting longer than 3 months
2	Presence of at least two additional visual symptoms from the following: a) Palinopsia: afterimages or trailing of moving objects b) Photophobia c) Nyctalopia (impaired night vision) d) Other persistent positive visual phenomenon including (but not limited to): enhanced entoptic phenomena (excessive floaters or blur field entoptic phenomenon), kaleidoscope type colours with eyes open or closed, spontaneous photopsias
3	Symptoms are not consistent with typical migraine visual aura
4	Symptoms are not better explained by another disorder

VSS is becoming increasingly self-diagnosed by our patients thanks to online search tools, and they will attend clinics to confirm this diagnosis. Previously this phenomenon was often dismissed by doctors as being psychological leading to increased patient anxiety. However, we now need to be careful that we are not making what would seem to be an easy diagnosis of VSS, when actually we are missing an underlying ophthalmic or neurological condition. This is an outline of how VSS is managed in my clinic, though others may prefer a different approach.

## Patient demographics

The population prevalence of VSS in the United Kingdom has been estimated to be 2% [3]. There have been reports of VSS from countries across the world, showing the phenotype and migraine comorbidity are remarkably similar in Australia [4], Italy [5], America [6], Israel [7] and Korea [8].

The average age at presentation is 29 years, though nearly 40% report having symptoms since childhood. There was no sex prevalence found in one large cohort [9], however, other papers have shown a female-to-male ratio of 1.6:1 [3], and in another 75% of VSS patients were male [4].

Typically, symptoms become disturbing enough to seek medical attention while doing final school exams, university exams or when the visual demands of work on a computer screen increase. Patients may attribute symptom onset to the time of a severe migraine attack. The prevalence of migraine and migraine with typical aura in VSS patients is higher than the general population at 50–80% [9, 10]. Some patients report a clear event trigger such as head injury, illness or infection including SARS-CoV2 [9, 11].

## Patient history

Patients with VSS describe seeing continuous, dynamic, tiny dots flickering across their entire visual field in both eyes. They might report this as being like the static or the ‘snow’ on a poorly tuned analogue television. This ‘snow’ is superimposed on the visual scene and while there is no loss of visual acuity nor a visual field defect, the patients may report that it interferes with their vision [9, 12]. Typically, the static is black and white, but it can be coloured. I would be cautious if the patient gave a story of sudden onset or if they were of older age at onset (see Table 2).

**Table 2:** Features on history and examination that would not be consistent with visual snow syndrome.

	Features suggestive of alternative pathology
History	- Sudden onset - Older age at onset - Static only in a small area of visual field or only in one eye - Associated drug use - Persistent bright positive phenomena with the eyes closed - Extreme nyctalopia or photophobia
Examination	- Reduced vision or visual fields - Pigmentary retinal changes - Ellipsoid zone changes on OCT macular raster images - Changes on FAF imaging

Other associated symptoms may be volunteered by the patient. Like with all history taking, I try to be as open in my questions as possible, rather than suggesting symptoms. I will ask if symptoms change depending on the time of day or where they are – to elicit nyctalopia or photophobia. I will also ask if there are ‘other things’ in their vision or if images persist in their vision after they have looked away. Persistent shimmering in the visual periphery when the patients’ eyes are closed would not be typical in VSS. Extreme nyctalopia and photophobia would also raise concerns for a retinopathy. Isolated entopic phenomenon without the presence of constant static could not classify as VSS.

It is important to take a history of migraine symptoms and any associated visual aura. Migraineurs share some of the symptoms experienced by VSS patients, such as photopsias and photophobia. While many of the migraine features overlap with VSS [13], visual phenomena are not directly linked to migrainous events and the description of visual snow is clearly distinct from their typical migraine auras [14, 15]. However, VSS symptoms are often more severe in individuals with a co-existing history of migraine [9, 16].

Mental health issues are often co-existing including anxiety and depression. Change in psychiatric treatment can be seen to exacerbate the symptoms, or equally treatment may have been beneficial. A social history looking at drug use is particularly important to differentiate VSS from hallucinogen persisting perception disorder (HPPD).

## Examination

Patients should have normal acuity for distance and near, as well as full visual fields. I typically will perform a Humphrey Visual Field 24-2 fast protocol.

Retinal mimics of VSS are typically visible on detailed optical coherence tomography (OCT) or fundus autofluorescence (FAF). It is important to look at the detailed macular raster images on the OCT for changes in the ellipsoid zone (see Table 2).

## Differential diagnosis

There are a few key differential diagnoses to consider in patients with symptoms otherwise suggestive of visual snow.

### Hallucinogen persisting perception disorder

HPPD represents spontaneous recurrence of visual perceptual disorders separated in time from the initial exposure to the hallucinogen. This condition has been reported in 5–50% of individuals exposed to lysergic acid diethylamide (LSD) and can last up to 5 years and probably beyond [17, 18]. Other ‘classic’ hallucinogens include psilocybin (magic mushrooms), mescaline and N-dimethyltryptamine. The atypical hallucinogens which have also been associated with HPPD include; 3,4-methylenedioxymethamphetamine, ketamine, phencyclidine and specific muscarinic agonists like scopolamine [19]. Delayed visual symptoms have also been reported following cannabis, often triggered by subsequent ethanol consumption or anaesthesia [20]. The recurrent hallucinations of HPPD may take the form of geometric shapes, objects in the peripheral vision, flashes of different colours, trail phenomena, afterimages, stroboscopic perception of movement, and/or disorders of size perception. HPPD patients tend to be older when compared to those with VSS. Over 80% can pin-point a specific onset of symptoms which would be unusual in VSS [9]. In a recent review of HPPD, none of the patients reported migraine, compared to more than half of VSS controls [21].

### Retinopathy

Visual snow or an increased awareness of photopsia has been described in patients with retinitis pigmentosa, but these patients also had reduced central acuity [22]. A case of birdshot chorioretinopathy has also been described, with the patient reporting a constant flickering disturbance in the vision misdiagnosed as VSS until an ERG was performed [23].

Patients with retinopathy and photoreceptor dysfunction from autoimmune conditions and cancer associated retinopathy present with photophobia and positive visual scintillations that are often described as gold or silver shimmering in the visual periphery. The shimmering is most noticeable in the dark and persists with the eyes closed. A careful history can usually differentiate this from VSS.

A case series of patients with glycine receptor autoimmunity describes symptoms of visual snow, palinopsia and positive visual phenomena which may be due to dysregulation of the GlyRα1 inhibitory neurotransmitter in the human retina [24].

### Charles Bonnet syndrome

Charles Bonnet syndrome (CBS) hallucinations can be characterised as simple flashes, dots of light or palinopsia. This phenomenon occurs in 40–60% of patients with profound bilateral loss of vision such as glaucoma or AMD [25]. However, examination will reveal these changes.

### Neurological disease

Any neurological abnormality of the visual cortex has the potential to trigger the perception of visual snow. This could include stroke, epilepsy, multiple sclerosis, neoplastic disease and degenerative diseases which should be apparent on history [6]. Episodic visual snow may occur in migraine and occipital lobe epilepsy but would not fulfil the diagnostic criteria for VSS [26]. There is a case report of a 55 year old man with 15 months of visual snow across his whole visual field, poor night vision and photopsias. Initial ophthalmic examination was normal. However, over subsequent months he developed spatial disorientation, myoclonus and ataxia. Neuroimaging showed occipital cortical ribboning and he was diagnosed with Heidenhain variant of Creutzfeldt–Jakob disease on brain biopsy [27].

## Investigations

In most cases, further investigations are not required when there is a typical history and normal physical examination. However, given that for many general ophthalmologists the diagnosis of VSS and the recognition of the classic syndrome compared to the potential ‘red flags’ (see Table 2) may not be simple, a case could be argued that ERG and MRI are required as part of a diagnosis of exclusion [28].

### When to consider blood tests

Blood tests would be reasonable to perform if there has been a sudden change in the patient’s symptomatology. Intercurrent illness can exacerbate the perception of VSS, so looking for signs of anaemia, infection, thyroid dysfunction or nutritional disturbance with blood tests could be requested. In first presentation of long-standing visual snow, I do not typically request blood tests, though I always encourage regular check-ups with the family physician.

### When to consider electrophysiology

If there is concern about an underlying retinopathy diagnosis is based on history, FAF or OCT, then proceed to electroretinography. However, electrophysiology is not required in majority of cases.

### When to consider neuroimaging

I do not routinely perform neuroimaging if the patients have had symptoms for a long duration, meet the diagnostic criteria and have normal visual fields. If the symptoms are very new onset or if there are visual field changes, then I request an MRI of the brain with gadolinium. While VSS is a clinical diagnosis, it is important that we remember that the final part diagnostic criteria is that the ‘symptoms are not better explained by another disorder’. Normal neuroimaging can be a source of great relief for anxious VSS patients. However, there is a chance of detecting normal variants or incidental changes which can lead to further unnecessary anxiety.

The diagnostic evaluation of VSS can be nuanced, and the diagnosis should be made on a case-by-case basis; however, if the VSS originated at an early age, is non-progressive and typical in historical presentation, the patient has a normal neuro-ophthalmic examination including normal perimetry and macular OCT; then, ancillary testing is generally unnecessary [28].

## Management

In general, VSS is non-progressive, though it does fluctuate in severity within and between patients. I will reassure the patients that VSS does not cause blindness and is not associated with any form of dementia. An explanation with reassurance and acknowledging the data and the range of presentations is a good place to start [29]. An honest discussion of the current state of research offers clarity and understanding which the patients generally find helpful. Some patients who are more prone to introspection and anxiety can struggle if they become too enmeshed in patient-led chat groups, so caution should be advised. However, others find the support of patients experiencing similar symptoms to be helpful [1].

In cases where the visual snow was brought on by an inciting event such as concussion, or infection, management of the underlying cause may significantly alleviate the VSS [6].

Colorimetry assessment for tinted lenses allows for a range of hues, saturation, and luminance to be tested while a patient looks at a visual target. Patients with VSS have been shown to respond positively to coloured filters within the blue–yellow spectrum, reporting a subjective improvement in their symptoms [4, 30].

Studies report partial improvement of VSS symptoms in some patients with drugs including, lamotrigine, topiramate, benzodiazepines and acetazolamide [11]. Others report only lamotrigine gave some improvement, but in a minority of patients [31]. In a large review of the 44 medications tried in VSS, only 8 were effective at least once; lamotrigine, topiramate, valproate, propranolol, verapamil, baclofen, naproxen and sertraline. The best data were available for lamotrigine being effective in 8/36 (22%) cases, followed by topiramate being effective in 2/13 (15.4%) [32]. However, I have not found any of these drugs to be helpful, and the side effects are often worse than the VSS.

Psychiatric symptoms are highly prevalent in patients with VSS and are associated with increased visual symptom severity and reduced quality of life [33]. Therefore, treatment of psychiatric symptoms can offer an avenue for clinician to help the patients improve their quality of life and ability to cope with other symptoms. However, in a review of patients using serotonin reuptake inhibiting antidepressants, 8.9% reported visual snow, 10.5% palinopsia, 15.3% photophobia and 17.7% nyctalopia as a side effect of the medication [34]. Amitriptyline [32] and citalopram [35] have also been reported to cause a worsening of VSS.

Repetitive transcranial magnetic stimulation applied to the visual cortex and compared to sham treatment in VSS patients resulted in a trend to subjective improvement of visual snow intensity [36]. However, it has not been widely tested or used and measures of treatment success also need to be determined before large scale trials of any intervention are started.

In our experience, for some patients, visual snow is extremely debilitating and results in behaviour like that seen with chronic disease. For these severe cases assistance, reassurance and managing other underlying issues can help them complete their schooling or participate actively in the workforce.

In my clinic, I explain that the symptoms will vary from day to day and month to month. They should be encouraged to manage any associated migraine or mental health issues with their family physician or a specialist. A deterioration in VSS symptoms might be a sign that they have other health issues or other stressors in their life and encourage them to again seek family physician review and mental health support if this happens. I encourage patients to participate in some relaxing activity that they enjoy such as meditation, yoga or just going for a regular walk at the end of the day; anything to reduce the tendency to anxiety and hypervigilance. There is some new work showing that programme of mindfulness-based cognitive therapy improved symptoms in VSS patients [37]. I always offer patients follow-up, so they do not feel dismissed. However, I do not routinely schedule a visit, but rather leave the offer open.

More patients with visual snow will be attending clinic, as the increase online resources means patients will self-diagnose and they will have questions about proposed therapies which are also being touted online. Fortunately, there is a lot of new research in this area, with potential therapies being the next target.
